# Deciphering Genome Content and Evolutionary Relationships of Isolates from the Fungus *Magnaporthe oryzae* Attacking Different Host Plants

**DOI:** 10.1093/gbe/evv187

**Published:** 2015-10-09

**Authors:** Hélène Chiapello, Ludovic Mallet, Cyprien Guérin, Gabriela Aguileta, Joëlle Amselem, Thomas Kroj, Enrique Ortega-Abboud, Marc-Henri Lebrun, Bernard Henrissat, Annie Gendrault, François Rodolphe, Didier Tharreau, Elisabeth Fournier

**Affiliations:** ^1^INRA, UR 1404, Unité Mathématiques et Informatique Appliquées du Génome à l’Environnement, Jouy-en-Josas, France; ^2^INRA, UR 875, Unité Mathématiques et Informatique Appliquées de Toulouse, Castanet-Tolosan, France; ^3^INRA, UR 1164, Unité de Recherche Génomique Info, Versailles, France; ^4^CNRS, UMR 8079, Ecologie, Systématique et Evolution, Université Paris-Sud, Orsay, France; ^5^Center for Genomic Regulation, Barcelona, Spain; ^6^INRA, UMR 385, Biologie et Génétique des Interactions Plantes-Pathogènes BGPI, INRA-CIRAD-Montpellier SupAgro, Campus International de Baillarguet, Montpellier, France; ^7^CIRAD, UMR 385, Biologie et Génétique des Interactions Plantes-Pathogènes BGPI, INRA-CIRAD-Montpellier SupAgro, Campus International de Baillarguet, Montpellier, France; ^8^INRA-AgroParisTech, UMR 1190, Biologie et Gestion des Risques en Agriculture BIOGER-CPP, Campus AgroParisTech, Thiverval-Grignon, France; ^9^Architecture et Fonction des Macromolécules Biologiques, Université d’Aix Marseille, France; ^10^Department of Biological Sciences, King Abdulaziz University, Jeddah, Saudi Arabia

**Keywords:** adaptation to the host, rice blast, comparative genomics, http://genome.jouy.inra.fr/gemo

## Abstract

Deciphering the genetic bases of pathogen adaptation to its host is a key question in ecology and evolution. To understand how the fungus *Magnaporthe oryzae* adapts to different plants, we sequenced eight *M. oryzae* isolates differing in host specificity (rice, foxtail millet, wheat, and goosegrass), and one *Magnaporthe grisea* isolate specific of crabgrass. Analysis of *Magnaporthe* genomes revealed small variation in genome sizes (39–43 Mb) and gene content (12,283–14,781 genes) between isolates. The whole set of *Magnaporthe* genes comprised 14,966 shared families, 63% of which included genes present in all the nine *M. oryzae* genomes. The evolutionary relationships among *Magnaporthe* isolates were inferred using 6,878 single-copy orthologs. The resulting genealogy was mostly bifurcating among the different host-specific lineages, but was reticulate inside the rice lineage. We detected traces of introgression from a nonrice genome in the rice reference 70-15 genome. Among *M. oryzae* isolates and host-specific lineages, the genome composition in terms of frequencies of genes putatively involved in pathogenicity (effectors, secondary metabolism, cazome) was conserved. However, 529 shared families were found only in nonrice lineages, whereas the rice lineage possessed 86 specific families absent from the nonrice genomes. Our results confirmed that the host specificity of *M. oryzae* isolates was associated with a divergence between lineages without major gene flow and that, despite the strong conservation of gene families between lineages, adaptation to different hosts, especially to rice, was associated with the presence of a small number of specific gene families. All information was gathered in a public database (http://genome.jouy.inra.fr/gemo).

## Introduction

Fungi attacking plants represent a major concern for food security and agriculture leading to important yield and postharvest losses, food quality alterations, and indirect environmental impacts. Fungi are currently considered as a major threat to global food security, with more than 125 million tons of the top five food crops (including rice and wheat) destroyed by fungi every year (Fisher et al. 2012; EASAC 2014). Numerous studies have shown that new fungal populations or species frequently evolve through host jumps (the new host is distantly related to the current host), host-range expansion (the new host is closely related to the current host), or host tracking (the divergence of the pathogen follows the divergence of the host, e.g., during host domestication) (Couch et al. 2005; Stukenbrock et al. 2007; Zaffarano et al. 2008; Frenkel et al. 2010; Silva et al. 2012). These mechanisms have caused the emergence of new fungal species responsible for important diseases of plants and animals (Fisher et al. 2012), highlighting the role of adaptation to a novel host as a driving force of population and species differentiation (Anderson et al. 2004; Giraud et al. 2010; de Vienne et al. 2013; Gladieux et al. 2014).

Deciphering the genomic bases of adaptation of fungal pathogens to new host(s) is key information for predicting the adaptive response of these organisms to global changes such as changes in landscapes and (agro)ecosystems, the emergence of diseases on new plants, the breakdown of plant resistances or the appearance of resistances to fungicide. This knowledge is crucial to enhance the durability of crop resistance to diseases, and is an active field of current research. In the last decade, genomic studies have boosted our understanding of adaptive mechanisms that allow fungi to rapidly adapt to new hosts, or more generally to contrasted environments (reviewed in Gladieux et al. 2014). One major result, emphasized by several studies, is the considerable fluidity of fungal genomes, that can be subject to genome expansions or contractions (Raffaele and Kamoun 2012), chromosome reshuffling (de Jonge et al. 2013), gain/loss of dispensable chromosomes (Ma et al. 2010; Croll et al. 2013) or genes (Galagan et al. 2005; Wapinski et al. 2007; Sharma et al. 2014), expansion of particular gene families (Haas et al. 2009; Duplessis et al. 2011), and horizontal gene transfers (Fitzpatrick 2012; Gardiner et al. 2012; Cheeseman et al. 2014). Adaptive evolution was also shown to rely on accelerated molecular evolution of particular genomic regions (e.g., Aguileta et al. 2010; Stukenbrock et al. 2011; Wicker et al. 2013) and on differences in gene expression (Fraser et al. 2012). In addition, transposable elements were shown to be important determinants of fungal genome evolution (drivers of macro- or microrearrangements, inducers of point mutations in surrounding coding or noncoding regions, or modifiers of gene expression; Beare et al. 2009; Rouxel et al. 2011; Grandaubert et al. 2014). Fungal gene families experiencing adaptive evolution include components of signaling pathways, structural proteins, receptors involved in environment sensing, secondary metabolism enzymes, secreted enzymes including cell-wall degrading enzymes, and small secreted proteins (SPs) (Xu et al. 2006; Soanes et al. 2007, 2008; Oliver 2012). Which of these processes act at small (intraspecific) evolutionary scale remains an open question.

The present genomic study was designed to identify the determinants associated with adaptation to the host using the fungal plant pathogen *Magnaporthe oryzae* as a model. This Ascomycete fungus is responsible for the rice blast disease, and is by far the most important fungal pathogen of rice, because of its destructive potential, its large geographic distribution, and its potential to overcome host resistance. The species *M. oryzae* gathers several genetically isolated host specific lineages pathogenic on either weed or crops: Rice, wheat, barley, foxtail millet (*Setaria* spp.), goose grass and finger millet (*Eleusine* spp.), cutgrass (*Leersia* spp.), and switchgrass (*Panicum* spp.). Host jumps from *Setaria* to rice at the time of rice domestication and later from rice to weeds (*Leersia*, *Panicum*) have been hypothesized (Couch et al. 2005). The same study hypothesized that *M. oryzae* lineages adapted to different host plants have diverged with very limited gene flow, leading to a tree-like genealogy. Conversely, although rice isolates populations are clonal at a local scale in most areas, footprints of ancient recombination exist (Couch et al. 2005) and evidences of contemporary sexual reproduction were provided in some locations of South East Asia near the center of origin of the disease (Kumar et al. 1999; Saleh et al. 2012, 2014). *Magnaporthe oryzae* is a model species for the study of airborne crop pathogens, and classical and functional genetic resources are available, leading to the identification of a large number of pathogenicity determinants (Talbot 2003; Ebbole 2007; Wilson and Talbot 2009). *Magnaporthe oryzae* was the first whole-genome-sequenced fungal pathogen of plant, with the Sanger sequencing of the rice reference isolate 70-15 (Dean et al. 2005). Three other *M. oryzae* rice isolates were recently newly sequenced (Yoshida et al. 2009; Xue et al. 2012; Wu et al. 2015), and their genome structure and composition was very close to the 70-15 reference genome. This genome is enriched in genes coding for SPs, in particular candidate effectors that are suspected to be key elements during the infection process by manipulating plant defense or deriving plant metabolism to the pathogen’s benefit (Chen et al. 2012; Giraldo and Valent 2013; Zhang and Xu 2014). To date, eight of the nine molecularly characterized avirulence genes in *M. oryzae* are small SPs (Valent and Khang 2010). The *M. oryzae* rice isolate genome is also enriched in enzymes involved in secondary metabolite biosynthesis.

To better understand how *M. oryzae* has adapted to different hosts, and especially to rice, we gathered a unique data set of eight isolates pathogenic to different plants. We choose five rice isolates pathogenic on *Oryza sativa*, as representative of the genetic diversity of this lineage worldwide (Tharreau et al. 2009; Saleh et al. 2014). The three other isolates were, respectively, pathogenic to foxtail millet (*Setaria italica*), wheat (*Triticum* sp.), and goosegrass (*Eleusine indica*). We added one isolate of *Magnaporthe grisea*, a related (Couch and Kohn 2002) but not sister (Klaubauf et al. 2014) species of *M. oryzae*, pathogenic to crabgrass (*Digitaria sanguinalis*). These nine isolates were sequenced using both 454 and Illumina (Solexa) technologies. Here, we described the genome content of these isolates and performed a comparative and evolutionary analysis of these genomes enriched with the 70-15 reference genome. We compared transposable element families and gene content among isolates, especially genes from families involved in the plant–fungal interaction, and looked for genomic differences between lineages adapted to different hosts, especially between the rice and the nonrice lineages. We also reconstructed the evolutionary history of these ten isolates and searched for signals of reticulate evolution.

## Materials and Methods

### Sequencing and Genome Assembly

The nine isolates sequenced in this study belong to different host-specific lineages. The five *M. oryzae* rice isolates, chosen as representative of the three major genetic groups identified worldwide (Saleh et al. 2014), were FR13 (from France, sexual type Mat1.1, belonging to the genetic group containing unfertile strains sampled on *japonica* rice), PH14 (from the Philippines, sexual type Mat 1.2, belonging to the genetic group containing unfertile strains sampled on *indica* rice), GY11 (from French Guyana, sexual type Mat 1.2, belonging to the genetic group containing fertile strains), TH16 (from Thailand, sexual type Mat 1.2, belonging to the genetic group containing fertile strains), and TH12 (from Thailand, sexual type Mat 1.1, belonging to the genetic group containing fertile strains). The three *M. oryzae* nonrice isolates were US71 (from the United States, pathogenic to *Setaria italica*), CD156 (from Ivory Cost, pathogenic to *Eleusine indica*), and BR32 (from Brazil, pathogenic to *Triticum* sp.). The *M. grisea* isolate used as an outgroup was BR29 (from Brazil, pathogenic to *D. sanguinalis*).

All DNA extractions were performed using stocks of fungal isolates stored on filter paper at −20 °C, as described by Valent et al. (1986). These stocks were initially obtained after monospore isolation and growth on rice flour medium as previously described by Silué and Notteghem (1990). For six of the nine isolates (FR13, PH14, US71, TH12, TH16, and GY11), DNA extractions were performed as described in Dioh et al. (2000). DNA of the three other isolates were kindly provided by Marc-Henri Lebrun after extraction on cesium chlorid gradient.

All isolates were sequenced at the Génoscope (Centre National de Séquençage, Evry, France) using both Solexa and Titanium technologies. One simple Solexa_GAIIX run (75 bp) and two mate-pair Titanium runs (8 kb) were performed for each isolate. Assemblies were performed at the Génoscope using Newbler 2.6 assembler that allows combining both Solexa and Titanium information (454).

The genome sequence of the reference isolate 70-15, sequenced using Sanger technology (Dean et al. 2005), was added to the analyses. The genome size of the sequenced *M. oryzae* 70-15 strain (version 8) is 40.9 Mb, and includes 12,991 predicted genes. Transposons consist of 9.7% of the genome (Thon et al. 2006).

### Genome Annotation

#### Gene Annotation

Gene structural prediction was performed using the EuGene software (Foissac et al. 2008) to combine results from 1) three ab initio prediction softwares: EuGeneIMM (Schiex et al. 2001), SpliceMachine (Degroeve et al. 2005), and FGENESH (http://linux1.softberry.com/berry.phtml, an ab initio gene predictor); and 2) homology-based comparison (using BLASTX and BLASTN) against Uniprot, fungal proteins obtained from 30 public genomes, and ESTs (expressed sequence tags) databases. The EuGene software was trained to generate *Magnaporthe*-specific matrix and to optimize parameters, using a set of 300 *Magnaporthe* genomic/full-coding cDNA pairs manually curated. Functional annotation of the coding sequences predicted with EuGene was carried out using InterProScan 5 (Quevillon et al. 2005). In the fasta files extracted from the gff files generated by the EuGene pipeline, genes were tagged as “Truncated” if they lacked start or/and stop codons. We used these tags to count truncated genes in each genome.

#### Burkholderia Filtering

*Bacterial* sequences were identified in four assemblies by both BLAST and GOHTAM (Menigaud et al. 2012) analyses. BLASTX analyses against NCBI (National Center for Biotechnology Information) nr protein database showed strong identity with *Burkholderia fungorum*. The detection and characterization of the sequences potentially originated from *Burkholderia* were performed by oligonucleotide usage profile discrimination and manual curation (see supplementary material S1, Supplementary Material online, for the detailed method).

#### Transposable Elements (TE) Annotation

TE were detected, classified, and annotated using pipelines from the REPET package (https://urgi.versailles.inra.fr/Tools/REPET). A library of consensuses representing TE families was first characterized on the reference genome of *M. oryzae* 70-15 using the TEdenovo pipeline (Flutre et al. 2011). Classification of consensuses was based on structural features (LTR, TIR, polyA queue, etc.) and on similarities using PFAM HMM domains (Sonnhammer et al. 1998) and the Repbase Update database (Jurka et al. 2005); we followed the unified classification system (Wicker et al. 2007). All the consensus TE sequences were manually reviewed and curated from chimeras, duplicates, and nested elements to build the TE reference library of *M. oryzae*. This library was then used to annotate the genome TE copies, even those nested and degenerated, on the ten genomes analyzed here using the TEannot pipeline (Quesneville et al. 2005).

#### Annotation of Genes Putatively Involved in Pathogenicity

SPs were predicted using a combination of classical predictors: SignalP 4.0 for peptide signals (Petersen et al. 2011) and TMHMM 2.0c for transmembrane domains (Krogh et al. 2001). The decision rules to predict a gene as SP-encoding were the following: 1) One predicted signal peptide ending at position 30 or less AND no predicted transmembrane domain OR, 2) one predicted signal peptide ending at position 30 or less AND one and only one predicted transmembrane domain ending at position 30 or less. To annotate genes involved in the biosynthesis of secondary metabolites in the nine de novo assemblies, we used the list of 48 such genes annotated in the reference 70-15 (Collemare et al. 2008) as queries for BLASTN and BLASTP search in the nine assemblies. Finally, carbohydrate-active enzymes were predicted using the CAZy database (Lombard et al. 2014). We restricted CAZy annotation to the six best assembled genomes (namely 70-15, TH16, US71, BR32, CD156, and BR29). Indeed the prediction of CAZymes is very sensitive to assembly quality, due to the modular nature of these genes.

#### Gene Family Prediction

Gene families were inferred between the predicted genes with OrthoMCL (Li et al. 2003) set with default parameters. The single-copy ortholog groups were defined from gene families including exactly one homolog gene in each of the ten compared genomes. Isolate-specific genes, defined as genes found only in one genome, were mostly single genes and were therefore not present in any groups of orthologs. Gene families composed of genes restricted to a single isolate were counted as isolate-specific genes.

#### Gemo Web Resource

All the annotation information acquired in this project is gathered and accessible through a web resource designed for the *Magnaporthe* community and available at http://genome.jouy.inra.fr/gemo. The database and Genome Browser (Gbrowse) system run on the international open source project Generic Model Organism Database (GMOD: http://www.gmod.org). The database was populated with the nine *Magnaporthe* isolates of the GEMO project along with their genome scaffold sequences as reference and several features predicted or mapped to these sequences: Predicted genes and associated functional annotations (Interpro, predicted SP, secondary metabolism, and CAZy) and annotated transposable elements. Bacterial regions from *Burkholderia* were also added in a dedicated track for the four concerned genomes.

### Evolutionary Analyses between the Ten Genomes

Each putative single-copy protein ortholog cluster recovered from orthology prediction was aligned using the T_Coffee software version 8.93 (Notredame et al. 2000). The corresponding nucleic alignment was obtained using the tranalign program of the EMBOSS package (Rice et al. 2000). We kept only ortholog clusters for which nucleic alignments were nonambiguous (this reduced the set of analyzed orthologs from 6,976 to 6,878). Aligned ortholog gene families were postprocessed to filter putative nonreliable positions using Gblocks (Castresana 2000).

#### Construction of a Reference Genealogy

Bayesian inference of individual phylogenetic gene trees was performed using the software MrBayes version 3.1.2 (Huelsenbeck and Ronquist 2001) on each of the 6,878 postprocessed aligned ortholog gene families. The evolutionary model was set to the HKY (Hasegawa–Kishino–Yano) with gamma-distributed rate variation across sites. The Bayesian analyses were carried out using two independent Markov chains and for 1,000,000 generations. The tree space was sampled every 1,000 generations to ensure at least 1,000 samples (1,000,000/1,000) for the estimation of posterior probabilities. The burnin fraction was set to 25% in order to summarize the trees and parameter values. All other MrBayes parameters were set to default values.

Bayesian Concordance Analysis (BCA) (Ané et al. 2007) was processed using the BUCKy software with default parameters (α = 1) (Larget et al. 2010). Individual Bayesian phylogenetic trees built for all single-copy otholog genes, served as input. The α parameter controls the a priori level of discordance among individual genes: α = 0 corresponds to the a priori assumption that there is no discordance among gene tree topologies (i.e., that all ortholog families have the same topology) and α = infinity corresponds to the a priori assumption that gene trees are completely independent (as in a consensus approach). α = 1 corresponds to the a priori assumption that about 50% of ortholog families share the same trees. BCA computes nonparametric clustering of genes with compatible trees and estimates the CF (concordance factors) for each lineage. The method reconstructs the primary concordance tree from lineages supported by the largest proportion of genes.

#### Network Reconstruction

To evaluate the presence of conflicting signals in the data set, and especially in the rice lineage, we built a distance matrix (F84 model) from the concatenation of the individual filtered alignments of the 6,878 putative orthologs. We excluded the BR29 *M. grisea* strain from this analysis, as well as the 70-15 isolate because of its hybrid origin; the alignments used for network reconstruction thus included eight *M. oryzae* sequences*.* We then apply the NeighborNet (Bryant and Moulton 2004) method implemented in the SplitsTree software (Huson 1998). The NeighborNet distance method is an extension of the Neighbor Joining algorithm allowing the representation of a network of conflicting signals in data, regardless of their sources.

#### Diversity Analysis and Recombination Detection

To detect recombination in the single-copy ortholog gene families and into the rice lineage, we used a simple and robust statistical test: The Pairwise Homoplasy Index (PHI) test (Bruen et al. 2006) based on detection of phylogenetically incompatible site-pairs. We used the PhiPack software, that also computes two diversity indices: The mean Pi values (i.e., the nucleotide diversity defined as the average number of nucleotide differences per site between any pair of DNA sequences randomly chosen from the sample) and the mean number of informative sites (IS) per alignment. A site of an alignment is informative only when it includes at least two different kinds of nucleotides, each of which is represented in at least two of the sequences under study. The PHI recombination test was applied on one hand to the 6,878 aligned orthologs, and on the other hand to 128 genomic aligned regions larger than 50 kb and present both in the 70-15 reference genome and in scaffolds of the rice isolates. These regions were obtained from a multiple genome alignment performed with the Mugsy software (default parameters) (Angiuoli and Salzberg 2011) and including the eight 70-15 scaffolds (used as reference genome) and all scaffolds of the five isolates of the rice lineage (see supplementary material S6, Supplementary Material online).

#### Estimation of the Fraction of 70-15 Genome that Belongs to the Rice Lineage

The phylogenetic analysis package ETE (Huerta-Cepas et al. 2010) was used to determine the proportion of different lineages found within the 6,878 topologies analyzed. Specifically, we looked for 1) the number of trees where 70-15 was placed within the rice lineage, 2) the number of trees where 70-15 was placed outside the rice lineage as a direct sister lineage, and 3) the number of trees where 70-15 was placed outside the rice lineage and was not a sister lineage to the lineage.

## Results

### Genome Sequencing and Assembly

Hybrid de novo assemblies of the nine *M. oryzae/M. grisea* genomes were performed independently for each isolate with Newbler 2.6. Resulting genome depths of coverage exhibited important variations (table 1*a*) ranging from 4 × (FR13 isolate) to 80 × (US71 isolate). Total assembly sizes (before *Burkholderia* filtering) varied between isolates: Six of the nine assembly sizes were comparable to the reference 70-15 reference strain (41–43 Mb) but three isolates (GY11, PH14, and TH12) exhibited higher assembly sizes of 46, 49, and 50 Mb, respectively (table 1*a*). Scaffolding was challenging for two genomes (FR13 and GY11) due to low yield of the sequencing process (FR13) or fragmentation of assembly (GY11, contig N50 < 9 kb). In brief, this new genome data set was somehow heterogeneous and comprised two highly fragmented genomes (FR13 and GY11), two medium-quality genomes (PH14 and TH12), and five high-quality assembled genomes (BR29, BR32, CD156, TH16, and US71) according to their assembly metrics.

**Table 1:** Assembly (*a*) and Annotation (*b*) Metrics for the Nine Analyzed Genomes Compared with the Annotation of the 70-15 Reference Genome, v8 Version (Available at the Broad Institute; values for 70-15 are in italics)

Isolate	*70-15*	FR13	GY11	PH14	TH12	TH16	US71	BR32	CD156	BR29
Host	*Oryza sativa*	*Oryza sativa*	*Oryza sativa*	*Oryza sativa*	*Oryza sativa*	*Oryza sativa*	*Setaria italica*	*Triticum* sp.	*Eleusine indica*	*Digitaria sanguinalis*
(*a*)										
Coverage	*—*	4×	42×	56×	48×	53×	80×	55×	50×	60×
Before *Burkholderia* filtering										
Total assembly size (Mb)	*40.9*	43.1	46.3	49.8	48.5	39.1	41.2	41.9	42.7	40.9
Total number of scaffolds	*8*	2,051	1,964	711	940	171	220	111	237	169
N50 of all scaffolds	*—*	101,645	187,276	697,115	590,463	938,544	813,981	1,760,460	1,066,457	955,448
%*N*	*0.2*	22.5	7.9	10.2	5.8	5.8	5.4	4.9	6.6	4.1
Total number of contigs	*—*	79,619	13,188	11,772	9,908	4,114	7,398	6,044	26,535	9,644
N50 of all contigs	*—*	1,726	8,565	17,330	20,191	30,676	42,462	27,863	29,891	43,743
After *Burkholderia* filtering										
*Magnaporthe* genome size (Mb)	*40.9*	42.4	39	40	40.1	39.1	41.2	41.9	42.7	40.9
Number of *Magnaporthe* scaffolds		1,924	632	496	347					
N50 of *Magnaporthe* scaffolds		103,882	226,076	756,803	715,884					
Genes—*ncRNA*	*—*	135	138	201	337	154	210	422	348	316
(*b*)										
Magnaporthe genes	*12,991*	14,384	14,781	13,816	14,026	13,571	13,803	14,336	14,067	12,283
Gene size (bp)	*2,008.1*	1,310.9	1,455	1,579.9	1,605.3	1,648.2	1,651.1	1,595.2	1,636.7	1,525.6
	*(1,435.7)*	(1,008.7)	(1,155.7)	(1,225.1)	(1,232.5)	(1,262.4)	(1,292.9)	(1,247.1)	(1,311.1)	(1,247.5)
CDS size (bp)		1,166.3	1,297.9	1,409	1,433.8	1,472.8	1,475.1	1,424.9	1,460.7	1,350.2
		(954.6)	(1,091.9)	(1,157)	(1,171.5)	(1,198.4)	(1,230.1)	(1,182.9)	(1,249.4)	(1,181.9)
Introns per gene		1.06	1.18	1.29	1.29	1.33	1.33	1.28	1.31	1.38
		(1.33)	(1.42)	(1.48)	(1.46)	(1.48)	(1.47)	(1.45)	(1.47)	(1.51)
Intergenic space (bp)	*1,414.7*	1,495.3	1,140.6	1,215.6	1,209.7	1,203.7	1,296.3	1,285.8	1,345.7	1,761
		(2,085.6)	(1,509.6)	(1,963.6)	(2,172.9)	(1,887.1)	(2,357.3)	(2,536.2)	(2,816)	(2,365)
Truncated genes	*225*	5,996	4,806	1,834	2,123	1,437	816	1,642	955	507
Truncated CDS size (bp)	*345.6*	916.4	918	948.6	966.2	916.1	788.7	969.7	875.3	786.2
	*(226.2)*	(868.7)	(958.1)	(1,066.3)	(1,131.5)	(1,045.4)	(1,107.3)	(1,188.5)	(1,155.7)	(870.6)
TE content (% gene without *Burkholderia*)	*11.1*	1.56	1.0	1.16	1.46	1.60	2.4	2.0	1.47	1.6

Note.—For FR13, GY11, PH14 and TH12, metrics are presented before and after *Burkholderia* filtering. In (*b*), mean gene, CDS, truncated and intergenic sizes are given for *Magnaporthe* genes only, with standard deviation between brackets.

Mitochondrial sequences were recovered in three of the nine genomes: BR29, BR32, and GY11. The sizes of cumulative mitochondrial sequences recovered in these assemblies were 42,451, 34,588 and 12,980 bp for BR29, BR32 and GY11, respectively. We attributed this discrepancy to the poor assembly of the GY11 genome. The 70-15 reference mitochondrion genome (supercontig 7.9 in the 70-15 v7 annotation: 34,953 bp) was recovered at 99% in the BR32 assembly but only at 37% in the GY11 assembly. The BR29 mitochondrial region was assembled in a single scaffold without gap, larger than the 70-15 reference mitochondrion, but covering only 68% of it. Mitochondrial regions and mitochondrial coding genes were filtered out for all further analyses.

### Filtering Out Nonfungal Sequences

In four of the nine genomes (FR13, GY11, PH14, and TH12), we detected unexpected large supplementary genomic regions almost identical to *B. fungorum* sequences (100% identity for 16S, recA, and gyrB genes), and therefore probably originating from one or several bacterial isolate(s) of this species. Genomic regions of bacterial origin were systematically filtered out using a parametric method based on genomic tetranucleotide signatures (Menigaud et al. 2012) followed by manual curation (supplementary material S1, Supplementary Material online). The cumulative sizes of *Burkholderia* regions were estimated to be 0.72, 7.26, 9.78, and 8.39 Mb in FR13, GY11, PH14, and TH12 assemblies, respectively (supplementary material S1, Supplementary Material online). Hereafter, we only focused on the *Magnaporthe* genomic and coding regions sequences.

**Fig. 1.—:**
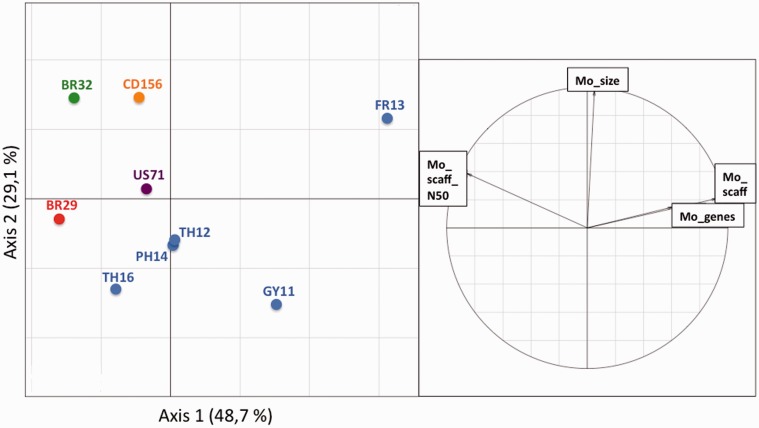
Comparisons of genome metrics, genome sizes, and gene content among isolates after *Burkholderia* filtering. The principal components analysis was performed for the nine de novo sequenced genomes on the following variables, recalculated after *Burkholderia* filtering when necessary: *Magnaporthe* genome size (Mo_size), number of *Magnaporthe* scaffolds (Mo_scaff), N50 of *Magnaporthe* scaffolds (Mo_scaff_N50), and number of *Magnaporthe* genes (Mo_genes). Colors indicate the host of origin (for *M. oryzae*, blue: rice, purple: foxtail millet, green: wheat, orange: goose grass; for *M. grisea*, red: crab grass).

After filtering out *Burkholeria* sequences, the quality of scaffolding of *M. oryzae* genomes exhibited important variations according to the scaffold numbers and the N50 values (fig. 1 and table 1*a*). The *M. oryzae* and *M. grisea* genome sizes ranged between 39 Mb (GY11) and 42.7 Mb (CD156) (table 1*a*). These differences were not correlated with the quality of the assemblies, as the second smallest (TH16, 39.1 Mb) and the largest one (CD156, 42.7 Mb) were among the best assembled genomes.

### Transposable Elements and Gene Content among Genomes and Lineages

Although TEs accounted for 11% of the 70-15 genome, the recovered TE proportions in the ten *M. oryzae/M. grisea* genomes ranged from 1% to 2.4% in the other *Magnaporthe* assemblies (table 1*b*). These differences were likely due to differences in sequencing methods and assembling between 70-15 (Sanger sequencing, Arachne assembly and finished genome) and the other genomes (454/Illumina sequencing, Newbler assembly, unfinished genomes). Twenty-eight TE families (37 TE consensus) found in the 70-15 genome were also detected in the other *M. oryzae* and *M. grisea* genomes. Most of TE consensuses were classified either as class I retrotransposons from the Gypsy superfamily (15 of 37 TEs) accounting for 45–70% of the TE space or as class II DNA transposons from Tc1/Mariner superfamily (9 of 37 TEs) accounting for 10–20% of TE space. We also annotated class I LINE (Long Interspersed Nuclear Elements) and SINE (Short Interspersed Nuclear Elements) accounting for 9–13% and 0.9–3.4% of TE space, respectively (supplementary material S2: table S2 and fig. S2*a*, Supplementary Material online). The distribution of TE families relative to TE space in the nine newly sequenced isolates (normalized with 70-15) showed that some families were over or underrepresented in the genomes of rice isolates (supplementary material S2: table S2 and fig. S2*b*, Supplementary Material online). Five families (3 class I LTR, 1 class I LINE, and 1 class II TIR) were overrepresented in isolates pathogenic to wheat (BR32), goosegrass (CD156), and crabgrass (BR29) and underrepresented in the ones pathogenic to rice. Although we possibly introduced a bias by using *M. oryzae* 70-15 genome as TE reference library to annotate other isolates, our results provide insights about the type and representation of transposable elements that invaded the different *Magnaporthe* genomes.

According to the EuGene de novo annotation, the gene contents varied from 12,283 genes (*M. grisea* BR29 isolate) to 14,781 genes (*M. oryzae* GY11 isolate). We found significant differences among the eight *M. oryzae* isolates (13,571–14,781 genes; χ^2 ^= 14, df = 7, *P* < 10^−^^5^) (table 1*b* and fig. 1*b*). Truncated genes were detected in all genomes and were in higher number in FR13, GY11, TH12, and PH14 isolates (table 1*b*). Significant differences in total gene numbers were also found between the four well-assembled *M. oryzae* genomes, that is, CD156, BR32, US71, and TH16 (13,803–14,336 genes; χ^2 ^= 23, df = 3, *P* = 3 × 10^−^^5^).

**Fig. 2.—:**
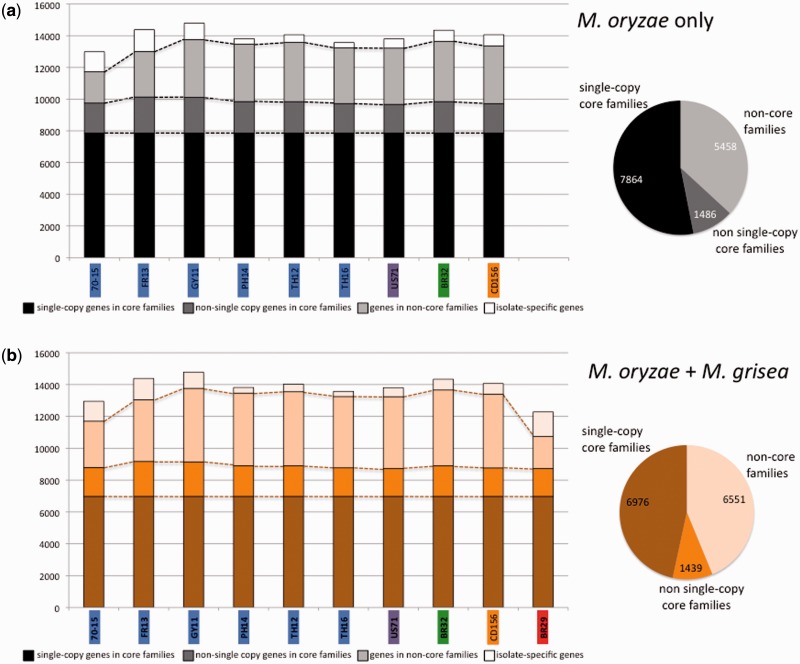
Organization of gene contents in the analyzed genomes, among the nine *M. oryzae* isolates (*a*) and among the whole data set (*b*). Histograms represent the distribution of genes in the different OrthoMCL families for each genome, and isolate-specific genes in white. Circles represent the proportion of OrthoMCL families. Colors on isolate names indicate host of origin (for *M. oryzae*, blue: rice, purple: foxtail millet, green: wheat, orange: goose grass; for *M. grisea*, red: crab grass).

Homology relationships between genes were predicted using OrthoMCL (Li et al. 2003) (fig. 2). The complete gene data set of gene prediction in the ten *M. oryzae* and *M. grisea* genomes included 138,058 fungal genes. It was organized into 8,089 isolate-specific genes, and 129,969 shared genes clustered into 14,966 OrthoMCL families comprising at least two genes from two distinct genomes. Among these gene families, 8,415 families (56.2 %) contained genes from all ten genomes, among which 6,976 families included only one copy in each genome, hereafter named single-copy orthologs (fig. 2*b*). In subsequent phylogenomic analyses, we analyzed a subset containing 6,878 of these single-copy orthologs (see Materials and Methods). We also estimated the number of OrthoMCL families shared among the nine *M. oryzae* isolates (fig. 2*a*). The *M. oryzae* set of shared genes was classified into 14,808 OrthoMCL families, among which 9,350 were shared by all *M. oryzae* isolates (7,864 with single-copy orthologs, 1,486 with nonsingle-copy orthologs).

### Annotation of Gene Categories Involved in Pathogenicity

To investigate differences between isolates with respect to adaptation, we examined the composition of each genome in categories of genes known to be involved in plant–fungal interaction: Genes involved in key steps of fungal secondary metabolism, carbohydrate-active enzymes, other secreted enzymes, and candidate effectors (table 2).

**Table 2:** Gene Contents of the Nine Newly Sequenced Isolates According to Specific Categories Involved in Pathogenicity

	70-15	FR13	GY11	PH14	TH12	TH16	US71	BR32	CD156	BR29
Genes in shared families	11,789	13,047	13,762	13,464	13,602	13,266	13,246	13,673	13,423	10,778
Secondary metabolism	48 (0.37)	91 (0.63)	90 (0.60)	75 (0.54)	84 (0.60)	61 (0.44)	63 (0.46)	89 (0.62)	73 (0.52)	63 (0.51)
		49^a^ (0.34)	45^a^ (0.30)	50^a^ (0.36)	48^a^ (0.34)	45^a^ (0.33)	48^a^ (0.35)	52^a^ (0.36)	52^a^ (0.37)	43^a^ (0.35)
CAZy	541 (4.2)					547 (4.0)	542 (3.9)	574 (4.0)	554 (3.9)	557 (4.5)
						515^a^ (3.8)	513^a^ (3.7)	539^a^ (3.8)	524^a^ (3.7)	529^a^ (4.3)
Other enz. SP	223 (1.72)	213 (1.48)	232 (1.57)	224 (1.62)	223 (1.59)	224 (1.65)	219 (1.59)	228 (1.59)	219 (1.59)	214 (1.74)
Non-enz. SP ≥ 300 aa	118 (0.91)	104 (0.72)	114 (0.77)	111 (0.80)	111 (0.79)	106 (0.78)	105 (0.76)	110 (0.77)	109 (0.77)	102 (0.83)
Non-enz. SP < 300 aa	38 (0.29)	32 (0.22)	39 (0.26)	37 (0.27)	41 (0.29)	38 (0.27)	38 (0.27)	39 (0.27)	38 (0.27)	37 (0.30)
Unknown SP ≥ 300 aa	508 (3.91)	560 (3.89)	587 (3.97)	545 (3.94)	551 (3.93)	510 (3.76)	522 (3.78)	543 (3.79)	531 (3.77)	499 (4.06)
Unknown SP < 300 aa	1,016 (7.82)	1,023 (7.11)	1,071 (7.26)	1,084 (7.87)	1,089 (7.78)	1,102 (8.12)	1,113 (8.06)	1,191 (8.31)	1,150 (8.17)	731 (5.95)
Isolate-specific genes	1,202	1,336	1,019	352	424	305	557	663	644	1,505
SP ≥ 300 aa	2	0	0	0	1	0	1	0	1	17
SP < 300 aa	68	74	70	19	24	16	33	31	39	137
Total genes	12,991	14,383	14,781	13,816	14,026	13,571	13,803	14,336	14,067	12,283

Note.—The percentages of each category based on the total number of genes in each genome are indicated between parentheses.

^a^Number of genes revised after manual BLAST-based curation.

Secondary metabolites play an important role in plant–fungal interactions (Collemare et al. 2008). In the 70-15 reference genome, previous studies identified 48 genes involved in key steps of secondary metabolism (PKS: polyketide synthase, NRPS: nonribosomal peptide synthase, hybrid PKS–NRPS or NRPS/PKS, TS: terpene synthase, DMATS: dimethylallyl tryptophane synthase). We searched for homologs of these 48 key genes in the ten *M. oryzae/M. grisea* genomes using a combination of BLAST searches, OrthoMCL families analyses, and phylogenetic tree construction. We identified a total set of 737 key genes involved in secondary metabolism (table 2 and supplementary material S3, Supplementary Material online). We identified instances where fragmentation in some genomes resulted in counting the same gene twice. Variation in copy number could therefore be due to fragmentation in some genomes. Manual reannotation using BLAST-based curation taking into account this fragmentation allowed reducing this set to 480 genes (table 2 and supplementary material S3, Supplementary Material online). The mean percentage of “false copies” of secondary metabolism genes was estimated to be about 35% across genomes. Fragmentation was clearly inflated in FR13 and GY11 genomes, as the percentage of false copies reached 46% and 50%, respectively. It might also be problematic in TH12 (43% of false copies) and BR32 (42% of false copies). In all other genomes, the percentage of false copies varied between 24% (US71) and 33% (PH14). After manual curation, the number of secondary metabolism genes was very close to the number in the reference genome, especially within the rice lineage. The lowest number was observed in the genome of the *M. grisea* isolate BR29 (43) and the highest number in the genomes of wheat and goose grass isolates BR32 and CD156, respectively (52).

Carbohydrate-active enzymes (CAZy) are secreted enzymes that catalyze the assembly and breakdown of complex carbohydrates and glycoconjugates (Lombard et al. 2014). They are modular proteins composed of catalytic modules and sometimes a noncatalytic carbohydrate-binding module. As the prediction of CAZy enzymes is sensitive to assembly quality, we performed this de novo prediction only for the best assembled genomes, that is, 70-15, TH16, US71, BR32, CD156, and BR29. In these assemblies, we identified a total set of 3,314 CAZy-encoding predicted genes (table 2 and supplementary material S4, Supplementary Material online). Again, we found that some genes were fragmented in some genomes and not in others, generating “false” copy number variation (e.g., the two GH45 of US71 are two fragments of a single GH45). This set could therefore be reduced to 3,130 genes after manual curation. The content in CAZy-encoding genes was highly conserved among genomes, varying from 541 (510 after curation) in 70-15 genome to 574 (539 after manual curation) in BR32 genome (table 2 and supplementary material S4, Supplementary Material online). We predicted 118 different types of catalytic modules, distributed as follows in 60 glycoside hydrolase types, 33 glycosyltransferase types, 10 carbohydrate esterases types, 11 auxiliary activity types, and 4 polysaccharide lyases types in addition to 16 noncatalytic carbohydrate binding module types. For half of the catalytic modules (53/118), the number of modules was identical in all six isolates. However, some families such as GH65, encoding a glycoside-hydrolase, were absent in two genomes (70-15 and TH16) whereas present in the other genomes.

Fungal SPs include putative effectors that manipulate host metabolism to facilitate infection (Stergiopoulos and de Wit 2009; de Jonge et al. 2011). After removing CAZy-predicted enzymes, we used 70-15 functional predictions to separate enzymatic from nonenzymatic and unknown SPs (table 2 and supplementary material S5, Supplementary Material online). The proportions of (non-CAZy) enzymatic SPs in the *M. oryzae* genomes were remarkably homogeneous, ranking from 1.48% in isolate FR13 to 1.65% in isolate TH16. The same trend was observed for nonenzymatic SPs, either large (over 300 residues) or small (below 300 residues), and for large unknown SPs. The marked differences were observed for small unknown SPs, whose proportions in *M. oryzae* genomes varied from 7.11% (FR13) to 8.31% (BR32). There were very few isolate-specific SPs (table 2).

Overall, this analysis showed that the different categories of genes involved in the interaction with the host were present in the same proportions in all *M. oryzae* and *M. grisea* genomes, without any particular expansion in the different host-specific lineages. Gene fragmentation was detected for specific gene categories (secondary metabolism genes) in some genomes, especially FR13 and GY11. In these two genomes, values of gene copy number in table 2 were inflated only for large unknown SP (respectively: +4.5% and +9.6% as compared with the other genomes) and for isolate-specific genes (respectively: +66.8% and +26.3% as compared with the other genomes), but clearly not for all gene categories.

### Evolutionary Relationships among the Ten Genomes

**Fig. 3.—:**
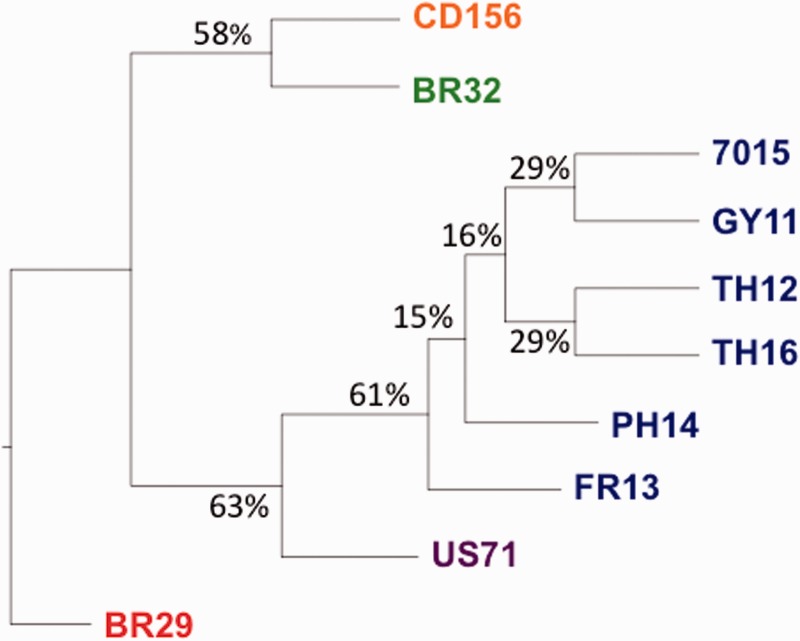
Primary concordance tree obtained from 6,878 *M. oryzae/grisea* single-copy orthologs. The primary concordance tree topology features relationships inferred to be true for the largest proportion of genes according the BUCKy software. CFs are indicated at internal branches and represent the proportion of individual gene phylogenies out of 6,878 in which this branch is resolved, providing a measure of branch support. Colors of leaves indicated host of origin of isolates (for *M. oryzae*, blue: rice, purple: foxtail millet, green: wheat, orange: goose grass; for *M. grisea*, red: crab grass).

We used an original, recently described method based on BCA (Ané et al. 2007) to analyze evolutionary relationships among the ten genomes. The BCA method allows estimating phylogenetic concordance among gene trees, starting from the collection of trees obtained from Bayesian analysis of each of the 6,878 single-copy gene families selected from the complete set of 6,976 single-copy orthologs. BCA combines all these individual-family analyses, using the full sample of trees from each family to account for individual gene tree uncertainty, without making any particular assumption regarding the reason for discordance among gene trees. Such discordance may arise from incomplete lineage sorting, horizontal gene transfer or hybridization, and was observed in our data set when using classical phylogenomics methods such as superalignment strategy or consensus supertree methods. The BUCKy software (Larget et al. 2010) was used to perform BCA and to analyze the primary concordance tree, which is composed of lineages supported by the largest proportion of genes. The topology of the primary concordance tree obtained from the 6,878 individual trees (fig. 3) was consistent with the biological knowledge we had about the ten analyzed isolates, especially genetic groups based on microsatellites (Tharreau et al. 2009; Saleh et al. 2014) and host specificity. All rice isolates clustered together as a monophyletic lineage sister to the foxtail millet lineage (US71). This relatedness confirms the result previously obtained by Couch et al. (2005) on the basis of ten nuclear genes. Within this rice lineage, we observed that, as expected, the 70-15 and GY11 isolates grouped together, an observation consistent with the genealogy of 70-15. This laboratory strain indeed originated from several complex in vitro crosses where GY11 was involved at least three times (Chao and Ellingboe 1991) (see below and fig. 5*a*). As expected, the two isolates from Thailand (TH12 and TH16) originating from the same population also grouped together. Outside of the rice lineage, the goose grass and wheat isolates (CD156 and BR32) appeared closely related to each other, which was quite unexpected according to previous data from Couch et al. (2005). Interestingly, CFs were quite low within the rice lineage (15–29%) compared with the other lineages (58–63%), showing that nodes outside the rice lineages were supported by at least half of the 6,878 individual gene trees, whereas a much lower proportion of individual gene trees supported nodes within rice lineage.

**Fig. 4.—:**
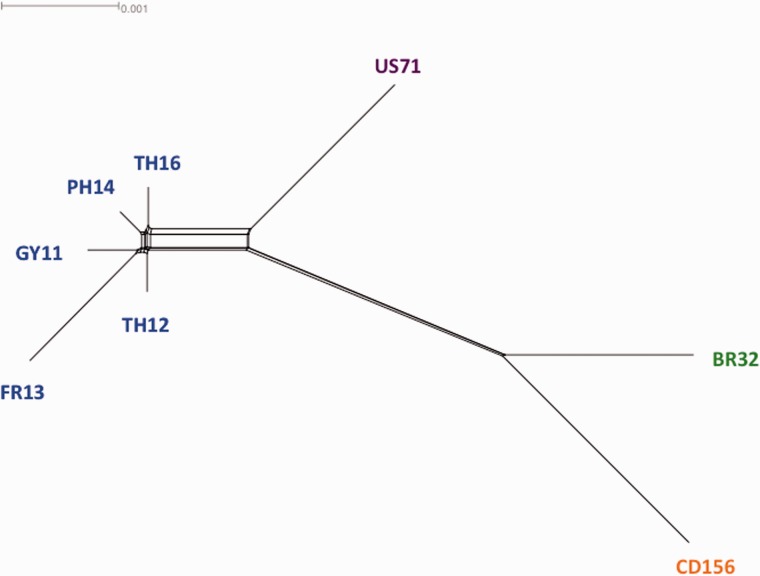
Network obtained from 6,878 *M. oryzae* orthologs. The NeighborNet method was applied to pairwise genetic distances (F84 distances obtained from the concatenated 6,878 orthologs aligned in codons). Colors of leaves indicated host of *M. oryzae* isolates (green: wheat, orange: goose grass, blue: rice, purple: foxtail millet). Isolates BR29 and 70-15 were not considered in this analysis.

To further investigate evolutionary history of the *M. oryzae* genomes, we also adopted a network representation, allowing the integration of different conflicting phylogenies. We applied the NeighborNet method (Bryant and Moulton 2004) implemented in the SplitsTree software (Huson 1998) on the matrix of pairwise genetic distances calculated from the concatenated alignment of the 6,878 orthologs, without taking into account the BR29 *M. grisea* isolate. We removed the reference genome 70-15 as it is a hybrid between rice and nonrice isolates. The resulting network (fig. 4) confirmed that conflicting signal existed in our data set, but indicated that it was mostly found within the rice lineage (GY11, FR13, TH12, TH16, and PH14 isolates). Conversely, evolution of the three nonrice isolates (US71, BR32, and CD156) was more supported by a tree-like topology, but conflicting signal was not completely absent along these nonrice branches. This analysis also confirmed the low divergence among rice isolates, as short branch lengths were observed within the rice network, except for FR13 (possible bias due to poor sequencing quality and low sequencing coverage of this genome).

### Estimation of Divergence and Putative Recombination within the Rice Lineage

To find out whether evidence of recombination existed among some of the nine *M. oryzae* genomes, we used the PHI test (Bruen et al. 2006) to detect possible traces of recombination in two data sets: 1) The 6,878 alignments of single-copy orthologs and 2) the 128 aligned genomic regions obtained from a whole-genome multiple alignment of scaffolds of the five rice isolates and the genome of the 70-15 strain (used as reference). Results confirmed that the nucleotide divergence was very low within the rice lineage (supplementary material S6, Supplementary Material online): The mean number of informative sites in the 6,878 alignments was ten times lower among the five rice isolates (0.53) than among all isolates (5.52). Similarly, the nucleotide diversity ranged between 0.21% and 0.63% among the rice isolates (for the 6,878 single-copy orthologs and the 128 genomic scaffold alignments, respectively) whereas it reached 5.52% when all isolates were considered. The PHI showed that footprints of recombination were negligible within the rice lineage, either in coding regions only (3 alignments with significant PHI signal out of 6,878) or in genomic alignments including both coding and noncoding regions (2 alignments with significant PHI signal out of 128).

### Footprints of Interlineage Hybridization in the 70-15 Genome

**Fig. 5.—:**
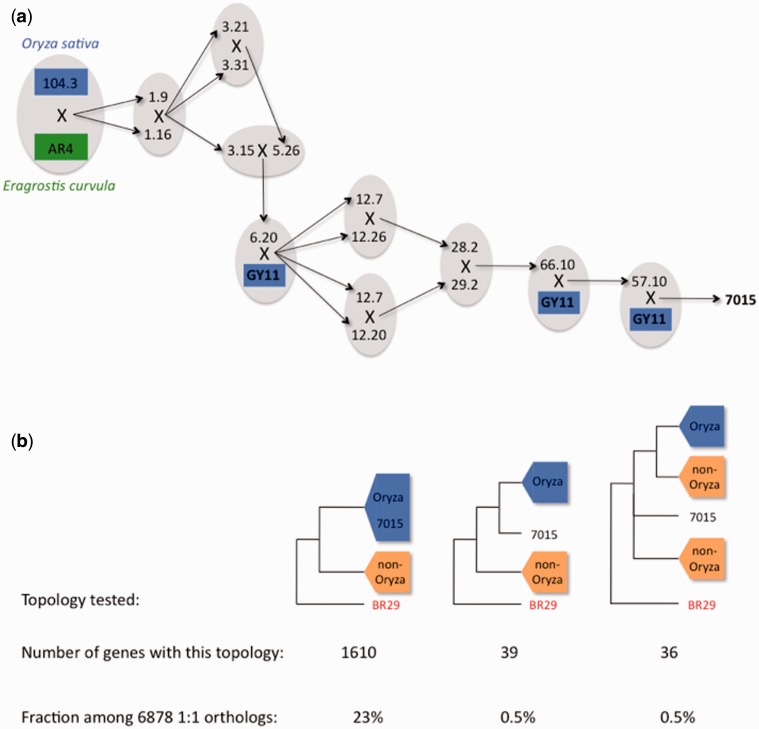
Genealogy of the 70-15 reference strain and estimation of the fraction of the 70-15 genome introgressed from nonrice lineage. (*a*) 70-15 was issued from a parental cross between a rice isolate and an *Eragrostis* isolate, followed by several crosses, three of them involving the GY11 rice isolate. X indicates in vitro crosses; arrows starting from X point toward offsprings of this cross. (*b*) Number and proportion of single-copy orthologs, among the 6,878 genes tested, exhibiting one of the three topologies where a rice lineage was fully resolved, and representative of three contrasted situations: 70-15 ingroup of the rice lineage, 70-15 outgroup and sister of the rice lineage, and 70-15 outgroup and not sister of the rice lineage.

As outlined above, the 70-15 reference strain is not a field isolate, and has a complex laboratory pedigree (Chao and Ellingboe 1991). It originates from an in vitro cross between a rice isolate and an *Eragrostis* isolate, followed by several backcrosses, three of them performed with the GY11 isolate (fig. 5*a*). We therefore expected footprints of hybridization between rice and nonrice lineages in the 70-15 genome. We used the ETE package (Huerta-Cepas et al. 2010) to estimate the proportion of genes among the 6,878 single-copy orthologs that support (or not) an assignment of 70-15 genome within the rice lineage. Out of the 6,878 topologies analyzed, only 1,676 topologies were recovered with a completely resolved rice lineage (grouping the five rice isolates in a monophyletic clade) (fig. 5*b*). In 1,610 of those 1,676 topologies (96%), 70-15 clustered together with the rice lineage, indicating that they shared a common ancestor. In 39 topologies (2% of the 1,676 trees with a resolved rice lineage), 70-15 formed a distinct but sister lineage of the rice lineage. Finally, in the 36 remaining topologies (2% of the 1,676 trees with a resolved rice lineage), 70-15 formed a distinct, but not sister lineage of the rice lineage. The latter cases correspond to genes for which 70-15 shares ancestry with a nonrice isolate. The nucleotidic divergence was 10-fold higher in these 36 genes (Pi = 0.012), confirming that they originated from a more distant lineage. These 36 genes did not cluster together in the same chromosome.

### Shared and Specific Families within and among Lineages

**Fig. 6.—:**
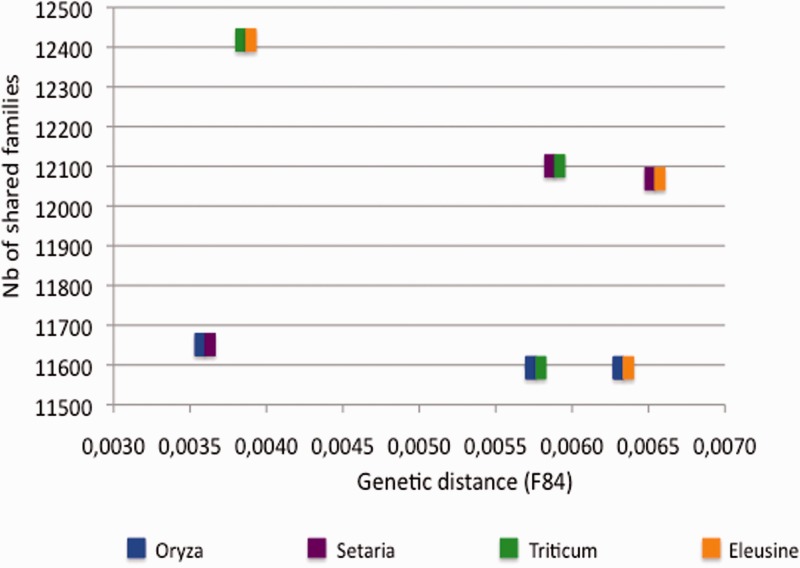
Number of OrthoMCL families shared among pairs of lineages as a function of the genetic distance between lineages. The F84 genetic distance between pairs of lineages was inferred from the Splitstree network (distance between the rice lineage and any other nonrice lineage: Mean distance between rice isolates and the corresponding nonrice isolate). The number of families shared between pairs of lineages was inferred from the OrthoMCL predictions (number of families shared between the rice lineage and any other nonrice lineage: Mean number of families shared between rice isolates and the corresponding nonrice isolate).

We further investigated whether the different host-specific *M. oryzae* lineages contained lineage-specific repertoires of genes. We performed all pairwise comparison of OrthoMCL families shared between strains belonging to different lineages. The number of shared families between rice lineage and any other lineage was calculated as the mean number of families shared between all rice isolates and the nonrice lineage. Results showed that the number of OrthoMCL families shared between two lineages did not correlate with the genetic distance between these lineages (fig. 6). Indeed, whatever the genetic distance between lineages, the rice lineage always shared fewer families with any other nonrice lineage than nonrice lineages among each-other. This indicated that the rice lineage contained a significant number of lineage-specific OrthoMCL families. To identify which category of genes could be responsible for these differences, we used OrthoMCL predictions to investigate how the different categories previously annotated were shared among lineages. To count OrthoMCL families shared by all *M. oryzae* isolates, we did not consider BR29 and authorized one missing gene in rice isolates. OrthoMCL families were considered as specific to nonrice isolates if they were shared by at least two nonrice isolates and absent of all rice ones. Conversely, OrthoMCL families were considered as specific to the rice lineage if they were shared by all rice-isolates but one and absent from all nonrice isolates.

We first looked at OrthoMCL families shared by all isolates and lineages. The distribution of OrthoMCL families containing key genes involved in secondary metabolism hardly differed among isolates and lineages. The 737 genes were distributed among 19 OrthoMCL families. Most of these families, that is, 17 of 19 (89.5 %) were shared by the nine *M. oryzae* genomes. A similar trend was observed for CAZy-encoding genes. Among the 557 OrthoMCL families containing CAZy, 494 (88.7 %) were shared by all *M. oryzae* isolates. We identified 205 OrthoMCL families containing non-CAZy enzymatic SPs, 96.6% of which were shared by all *M. oryzae* isolates. This was also the case for 92.4% of the 131 OrthoMCL families containing nonenzymatic SPs. Therefore, none of these categories (key genes involved in secondary metabolism, CAZy, enzymatic SP, nonenzymatic SP) was likely responsible for the observed differences in shared families among lineages. On the contrary, we found that unknown SPs were less shared by all *M. oryzae* isolates, as only 466 of 570 (81.7%) OrthoMCL families containing large SPs (≥300 amino acids), and only 876 of the 1,268 (69%) OrthoMCL families containing small SPs (<300 amino acids) were found in all nine *M. oryzae* isolates.

We identified 529 OrthoMCL families specific to nonrice isolates. Among them, we found a single secondary metabolism OrthoMCL family (after manual curation) restricted to the wheat and goose grass isolates (1 gene/assembly), 11 CAZy OrthoMCL families, and 8 OrthoMCL families containing large unknown SPs. Small unknown SPs represented a larger proportion of nonrice-specific OrhoMCL families, as we identified 69 such families, among which the avirulence gene *AVR1-CO39*.

Finally, there were 86 OrthoMCL families specific to the rice lineage. They contained nine SPs specific to the rice lineage, all small and including the avirulence gene *AVR-Pik*. The 77 other rice-specific OrthoMCL families contained two secondary metabolism groups (one PKS and one chalcone synthase), and no CAZy, other known enzymatic or nonenzymatic SPs. Based on the v8 annotation of the 70-15 strain, we could also identify two transcription factors and three cytochrome P450.

## Discussion

In this study, we compared the genomes of nine isolates from *M. oryzae* specialized on four different host plants, and one genome isolated from the closely related species *M. grisea*.

### Genome Content Only Slightly Differs among *M. oryzae* Isolates

Small differences in genome sizes (9.5% difference between the smallest assembly—GY11: 39 Mb—and the largest one—CD156: 42.7 Mb) and gene numbers (8.9% difference between the smallest assembly—TH16: 13,571 genes—and the largest one—GY11: 14,781 genes) were observed among the eight newly sequenced *M. oryzae* genomes. Such differences could be partly due to gene truncation, as highlighted for secondary metabolism genes. Truncated genes are due to either a low coverage of a specific region or a deficiency in the assembly process of the region. Indeed, most of these events were observed in FR13 and GY11 genomes that were poorly assembled. In addition, the number of isolate-specific genes was inflated in these isolates, which is another clue in favor of notable fragmentation in these genomes. However, fragmentation was restricted to some gene categories (secondary metabolism genes, large unknown SP, and isolate-specific genes) and was therefore not a general problem. Moreover, the observed differences in gene numbers could not be entirely attributed to gene fragmentation during assembly, as significant differences in gene numbers were also observed among the best assembled *M. oryzae* genomes (BR32, CD156, US71, TH16, with 5.6% difference between the smallest number of genes in TH16 and the largest one in BR32). Additional investigations are needed to test whether these differences are due to horizontally transferred DNA, as already observed for some genomic regions of *M. oryzae* isolates subject to merodiploidization (Böhnert et al. 2004), or to variation in dispensable chromosomes content among isolates (Talbot et al. 1993). Such karyotypic variability was also identified in other fungal species (Ma et al. 2010; Stukenbrock et al. 2010).

Still, the nine *M. oryzae* genomes had almost similar sizes and gene contents. Indeed, 70.5% of all genes were classified in OrthoMCL families. Especially, after manual curation, gene families corresponding to secondary metabolism genes were highly conserved among *M. oryzae* isolates. Similarly, secondary metabolism genes, and genes encoding CAZome, other known enzymatic and nonenzymatic SPs were also conserved among *M. oryzae* genomes, with very few isolate- or lineage-specific gene losses. Genome content of unknown SPs was more variable among lineages than the other categories. Overall, we have not observed drastic changes in gene content and gene family composition.

This situation differs from other fungi in which adaptive evolution relies on important genome fluidity, such as variation in genome sizes and gene numbers due to expansion of particular gene families, duplications or loss of large fragments, and horizontal acquisition of genomic material (Gladieux et al. 2014). Indeed, we showed that adaptation to different host plants in *M. oryzae* was apparently not associated with major modifications in gene content or gene family organization. In particular, the proportions of different categories of genes involved in pathogenicity were conserved among genomes.

### Phylogenetic Relationships among *M. oryzae* Isolates Estimated Using Whole Genomes

A phylogenomic analysis of these ten *M. oryzae*/*M. grisea* genomes showed that the rice-lineage diverged recently from the other lineages, and that reticulate evolution could be detected inside it. Among the different host-specific lineages, we also detected a weak signal of reticulate evolution. Based on 6,878 single-copy orthologs shared by all genomes, we showed reticulate (i.e., not tree-like) evolutionary relationships within the rice lineage, resulting in discordance among individual gene trees. These results were supported by both BCA analysis and network reconstruction. The low genetic divergence observed between rice isolates (average Pi of 0.002) could be responsible for the difficulties encountered building a tree using classical phylogenomics methods. We also found a weak signal of recombination within the rice lineage; this signal is however questionable as the level of DNA polymorphism is very low. Overall, according to our data set it is difficult to disentangle the possible sources of phylogenetic discordance within the rice lineage: Either recombination alone, or incomplete lineage sorting due to recent divergence, or horizontal gene transfers involving particular loci. To better resolve the evolutionary history within the rice lineage, there is a need for analyzing a larger number of isolates from this lineage using more polymorphic markers. Outside the rice lineage, we found that the wheat isolate (BR32) was closely related to the goose grass one (CD156), which was not expected according to Couch et al. (2005). This suggests possible conflicting phylogenetic signal among markers. Globally, our results confirmed that lineages adapted to different host plants have diverged from each other without important gene flow. However, only 60% of the 6,878 topologies supported a congruent topology outside the rice lineage, and the network reconstruction also confirmed the existence of reticulate evolution among host-specific lineages. This indicated probably ancient gene flow (hybridization or horizontal gene transfers) or incomplete lineage sorting among lineages. Deciphering the exact history of divergence among host-specific lineages requires phylogenomic analyses of a larger number of representative isolates from each lineage.

### Hybrid Origin of 70-15 Genome

The phylogenomic analysis performed also detected genomic footprints of the hybrid origin of the 70-15 isolate. We found that a small fraction (2%) of the 70-15 genome originated from nonrice isolate(s), which is in agreement with the crossing scheme leading to 70-15. However, this fraction was lower than what would be expected from the number of backcrosses involved. Indeed, 70-15 is a progeny originating from a cross between an *Eragrostis* isolate and a rice isolate followed by four backcrosses to rice isolates. Such a scheme should lead to a proportion of 6.25% of the rice isolate genome being introgressed from the *Eragrostis* isolate genome, which is three times higher than the observed proportion (2%). Two reasons may explain that the observed introgression rate was lower than expected. First, selection might have affected some regions during the crossing process, leading to their elimination, and second, the introgressed fraction could be far from expectation just by chance as only a single offspring was chosen for each backcross. This estimated introgressed fraction could have also been underestimated as we only used coding sequences from the core genome.

### Presence of Bacterial Sequences in Assemblies

Four of the nine genome assemblies contained unexpected bacterial sequences belonging to *B. fungorum*. A total of 2,291 scaffolds were attributed to *B. fungorum* (supplementary material S1, Supplementary Material online). Only 22 of these scaffolds (15 in the PH14 assembly, 7 in the FR13 assembly) were chimeric, containing sequences from both *Magnaporthe* and *Burkholderia*. In all such scaffolds, fungal and bacterial regions were separated by N stretches, suggesting scaffolding errors. The origin of this bacterial DNA, the conditions of its presence, and the potential impact of the presence of the bacteria on *Magnaporthe* phenotype or on its interaction with the host plant remain to be elucidated.

### Transposable Elements

TEs shape fungal genomes, either through their own integration/excision dynamics or through RIP-associated mutations (Amyotte et al. 2012; Santana et al. 2012; Grandaubert et al. 2014). In *M. oryzae*, TEs are also involved in telomere stability (Farman 2007; Starnes et al. 2012), and some avirulence genes have TEs in their immediate genomic surrounding (Valent and Khang 2010). The estimated genomic space occupied by transposable elements in the newly sequenced *M. oryzae*/*M. grisea* genomes was 10-fold lower than in the 70-15 reference genome. Highly repeated regions are generally poorly assembled with new sequencing technologies. Therefore, prediction of TE families in the nine de novo genomes analyzed here could be interpreted only qualitatively, or quantitatively relative to the TE space. Exact quantification of TE repeats in these different genomes would require alternative sequencing strategies.

### Rice Lineage-Specific Genes and Host Specificity

We found that isolates from the rice lineage shared a significant number of OrthoMCL families without representative in genomes of nonrice isolates. The 86 OrthoMCL families specific to the rice lineage corresponded only to two secondary metabolism families, no CAZy, no known enzymatic SP and few unknown SPs genes (nine families). In addition, more than 50% of the rice-lineage-specific OrthoMCL families had unknown functions and were not expressed during either mycelium growth or infection (Lebrun M-H, unpublished research). Hence, the adaptation to rice is likely associated with the occurrence of a small number of lineage-specific genes, among which unknown small SPs. This supports the view that host specificity in the *M. oryzae* lineages might rely on a small number of genes. Indeed, in a cross between a weeping lovegrass isolate and an isolate pathogenic to both weeping lovegrass and rice, Valent and Chumley (1991) obtained 2 of 59 F1 progeny (i.e., 3%) pathogenic on rice, a proportion equivalent to the segregation of five additive genes involved in host specificity. Other studies investigated the F1 progeny from genetic crosses between wheat and foxtail millet isolates (Murakami et al. 2000, 2003), wheat and wild oat isolates (Oh et al. 2000), or wheat and rice isolates (Tosa et al. 2006), identified from one to five major segregating genes involved in host specificity. Host specificity could also be controlled by the absence of few avirulence genes in a given lineage. One example of such avirulence-based host specificity is the loss of *AVR1-CO39* in all rice isolates, despite its conservation in all nonrice isolates (Cesari et al. 2013). Adaptation to host plant could also result from allelic variations in genes directly or indirectly involved in interaction with plants, such as regulatory genes or genes involved in signal transduction, or on specific expression profiles of some of these genes, possibly induced by polymorphisms from regulatory noncoding regions.

Several directions for future research could be proposed to test these hypotheses. First, by further characterizing the small differences of secondary metabolism and CAZy-predicted genes across genomes, it will be possible to address whether these little variations could potentially be important for adaptation to the host. Next, the analysis of potentially functional allelic variation across orthologs will allow testing whether particular gene categories are targeted by adaptive molecular evolution among host-specific lineages. The identification of sets of genes unique to the rice lineage but absent from nonrice ones could be further confirmed by additional complete sequencing of new isolates. Finally, genome-wide transcriptomic analyses in different conditions, especially at different infection steps, would help disentangling the respective roles of lineage-specific genes and lineage-allelic variants in adaptation to the host.

This study represents the first comparative and evolutionary analysis of several genomes of the fungal pathogen *M. oryzae* using isolates with different host plants specificities. This unique data set showed that adaptation to different host plants was not associated with major changes in gene content or gene family composition. The proportions of the different categories of genes involved in plant–fungal interaction were similar from one genome to another. However, we also identified 86 OrthoMCL families specific to the rice lineage that contained a small proportion (10%) of unknown small SPs. This study confirmed that *Magnaporthe* lineages diverged without important gene flow and suggested that only few genes are responsible for host specificity.

## Supplementary Material

Supplementary materials S1–S6 are available at *Genome Biology and Evolution* online (http://www.gbe.oxfordjournals.org/).

Supplementary Data
