# Supplementary material for: Deciphering Genome Content and Evolutionary Relationships of Isolates from the Fungus Magnaporthe oryzae Attacking Different Host Plants
Source: Genome Biol Evol. 2015 Oct 9;7(10):2896–912. doi: 10.1093/gbe/evv187 (PMC4684704; doi:10.1093/gbe/evv187)
Supplement: Supplementary Data [file supp_7_10_2896__index.html]

Deciphering genome content and evolutionary relationships of isolates from the fungus Magnaporthe oryzae attacking different host plants. — Deciphering Genome Content and Evolutionary Relationships of Isolates from the Fungus Magnaporthe oryzae Attacking Different Host Plants — Supplementary Data 

# Deciphering Genome Content and Evolutionary Relationships of Isolates from the Fungus *Magnaporthe oryzae* Attacking Different Host Plants

## Supplementary Data

files

- Supplementary Data - pdf file
- Supplementary Data - docx file
- Supplementary Data - xlsx file
- Supplementary Data - xlsx file
- Supplementary Data - xlsx file
- Supplementary Data - docx file
