# Supplementary material for: Deciphering Genome Content and Evolutionary Relationships of Isolates from the Fungus Magnaporthe oryzae Attacking Different Host Plants
Source: Genome Biol Evol. 2015 Oct 9;7(10):2896–912. doi: 10.1093/gbe/evv187 (PMC4684704; doi:10.1093/gbe/evv187)
Supplement: Supplementary Data [file supp_evv187_Chiapello-SupS1-revised2.pdf]

Supplementary S1. Method and results of *Burkholderia* filtering

Method

- **Setting of *Magnaporthe* and *Burkhloderia* prototypes**

To filter *Burkholderia* regions, we designed prototypes of the tetranucleotide usage of both *Burkholderia* and *M. oryzae* DNA present in our assembly as follows. The *Magnaporthe* prototype was learned from the longest scaffolds where no blast hit to *Burkholderia* species from the non-redundant division of GenBank could be found. The *Burkholderia* prototype was computed over the scaffolds exhibiting over 98% coverage from blast hits against *Burkholderia* species in GenBank nr. The prototypes of *Burkholderia* were learned independently on each of the 9 genomes assembly and computed from regions of least 100 Kb.

- **Genome assemblies scanning**

Genome assemblies were scanned independently with a sliding window system (5 kb long, 100bp step) respectively against both *Burkholderia* and *Magnaporthe* prototypes using a Kullback-Leibler (KL) divergence. The frequency distributions of the KL divergence across all windows were plotted for both prototypes and all assemblies (Figure S1a). This representation allows visualizing oligonucleotide composition similarity and quantity for all genome assemblies. Five out of the nine assemblies exhibited window distributions including only one peak corresponding to DNA from *Magnaporthe regions.* For four assemblies (FR13, GY11, TH12 and PH14) secondary peaks were detected corresponding to DNA with different oligonucleotide composition originated from *Burkholderia*.

- **Thresholds setting**

Thresholds for *Burkholderia* and *Magnaporthe* were manually set to best split the two main modes of the distribution when a second peak in distribution was detected (Figure S1a). To avoid false positives from the border effect of the thresholds, a double threshold system was used. Regions were labelled as *Burkholderia* when the following two conditions were met: (i) the KL divergence to the *Magnaporthe* prototype was over their respective threshold (1000 Arbitrary Units) for *Magnaporthe,* and (ii) the KL divergence to the *Burkholderia* prototype was smaller then the threshold for *Burkholderia* (930 AU).

- **Taxonomical assignment**

The sequences selected with this parametric method were analyzed with GOHTAM (Menigaud, et al. 2012) and confirmed the homogeneity and origin from Burkholderiales. Comparison of the selected DNA to the results of Blast used to learn the prototype of *Burkholderia* showed a significant increase in DNA coverage for the parametric method over Blast while the oligonucleotide composition homogeneity was conserved.

- **Manual curation**

This automatic detection of *Burkholderia* regions was followed by a manual curation. Scaffolds that comprised both *M. oryzae* and *Burkholderia* regions were systematically verified by Blastn and Blastx, and corrected when necessary. A *Burkholderia* tag was reported in corresponding OrthoMCL families for all genes located in *Burkholderia*

regions. Following this, some OrthoMCL families comprised both *Burkholderia* and *Magnaporthe* genes. In these cases, scaffolds containing these particular *M. oryzae* genes were verified by Blastn and Blastx, and corrected when necessary.

Results

Large supplementary genomic regions were confirmed in four out of the nine genomes (FR13, GY11, PH14 and TH12). The cumulative sizes of *Burkholderia* regions were estimated to be 0.72, 7.26, 9.78, and 8.39 Mb in FR13, GY11, PH14 and TH12 assemblies, respectively. *Burkholderia* scaffolds and regions (see table S1) were systematically tagged and filtered out of the assemblies for further bioinformatics analyses.

GOHTAM taxonomical assignment of these regions confirmed the homogeneity and origin from *Burkholderiales*. Interpretations from the results suggested an unsequenced species closely related to *Burkholderia phytofirmans* and *Burkholderia xenovorans*. Targeted Blast comparisons indicated that some of these supplementary regions are almost identical to *Burkholderia fungorum* sequences (100 % identity for 16S, recA and gyrB genes), and therefore probably originating from one or several bacterial isolate(s) of this species.

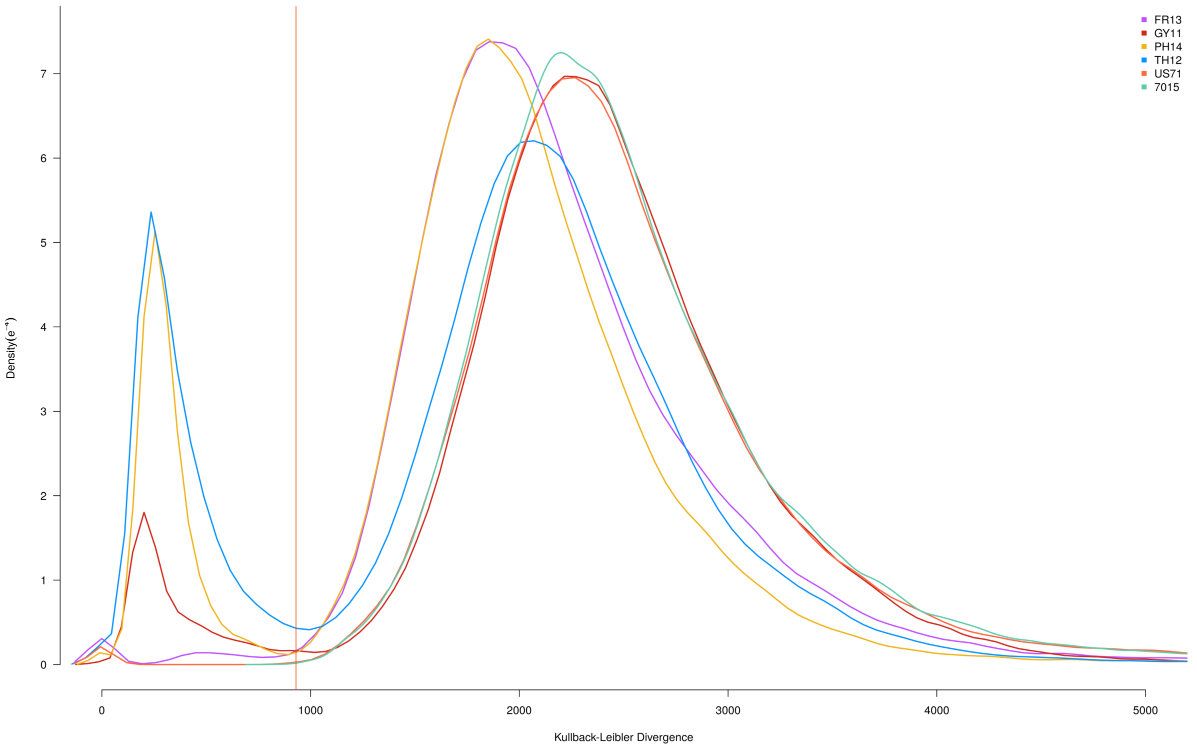

<u>Figure S1a: Distribution of the window compositional divergence according to the *Bukholderia* prototype</u>

Distribution of the Kullback-Leibler (KL) divergence per genome between the tetranucleotide composition of the sliding windows and the prototype tetranucleotide composition of the genome-specific *Burkholderia* sequences identified by Blast homology to *Burkholderia* species present in GenBank nr. *Burkhorderia* prototypes were learned per genome and on more than 100 Kb DNA. *Burkholderia* prototypes built from each genome were found very similar. The distribution of the KL from a fixed genome were comparable when the *Burkholderia* prototype from another genome was used. Compared to the composition of a *Burkholderia* prototype, low divergences are expected from sequences from *Burkholderia* or compositionally close. Hence a low value peak corresponded to *Burkholderia*, and a high value peak corresponded to *Magnaporthe* sequences. We used the strains 7015 and US71 as control to set the threshold at the junction of distributions.

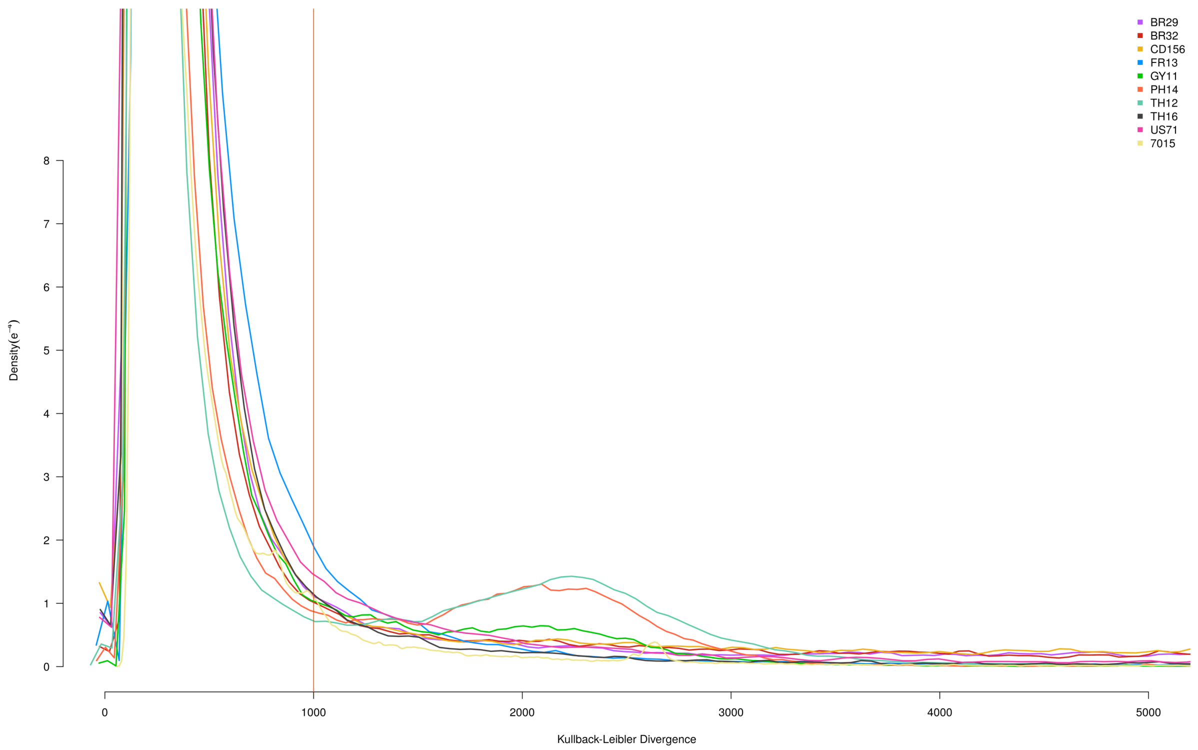

<u>Figure S1b: Distribution of the window compositional divergence according to the *Magnaporthe* prototype</u>

Distribution of the Kullback-Leibler (KL) divergence per genome between the tetranucleotide composition of the sliding windows and the prototype tetranucleotide composition of the genome-specific *Magnaporthe* sequences extracted from the assembly scaffold when Blast hits against GenBank nr. did not match *Burkhorderia* species for more than 1% coverage. All the scaffolds matching this criteria were used to build the prototype compositional profile. Compared to a composition of *Magnaporthe*, low divergences are expected from sequences of the fungal genome. The low value peak correspond to *Magnaporthe* sequences, and the second peak to *Burkholderia*.

<u>Table S1: Details on *Burkholderia* regions found in the four *M. oryzae* genome assemblies.</u>

Isolate	PH14	TH12	GY11	FR13
Burkholderia regions (Mb)<br>and % of total assembly size	9.78<br>19.6%	8.39<br>17.3%	7.26<br>15.7%	0.72<br>1.7%
Number of *Burkholderia* scaffolds	232	593	1332	134
Mean size of *Burkholderia* scaffolds in kb<br>(standard deviation)	43.1<br>(118.9)	14.1<br>(12.3)	5.5<br>(3.8)	5.3<br>(8.1)
Number of chimeric *Burkholderia* scaffolds	15	0	0	7
Number of *Burkholderia* genes	6050	5448	5690	516
