# Supplementary material for: Deciphering Genome Content and Evolutionary Relationships of Isolates from the Fungus Magnaporthe oryzae Attacking Different Host Plants
Source: Genome Biol Evol. 2015 Oct 9;7(10):2896–912. doi: 10.1093/gbe/evv187 (PMC4684704; doi:10.1093/gbe/evv187)
Supplement: Supplementary Data [file supp_evv187_Chiapello-SupS2.docx]

**Supplementary S2:** Transposable Element (TE) annotation

**Table S2: Annotation of transposable elements using 7015 TE consensus. TE content of class I and class II main orders and super families in bp.**

|  | ***Oryza*** | ***Oryza*** | ***Oryza*** | ***Oryza*** | ***Oryza*** | ***Oryza*** | ***Setaria*** | ***Triticum*** | ***Eleusine*** | ***Digitaria*** |
| --- | --- | --- | --- | --- | --- | --- | --- | --- | --- | --- |
|  | **7015** | **FR13** | **GY11** | **PH14** | **TH12** | **TH16** | **US71** | **BR32** | **CD156** | **BR29** |
| Class I LTR Copia | 513617 | 82400 | 67111 | 87341 | 126292 | 83834 | 93686 | 107743 | 98817 | 31230 |
| Class I LTR Gypsy | 2035925 | 344140 | 203731 | 247203 | 331048 | 375719 | 654722 | 415109 | 262135 | 327084 |
| Class I LTR other | 217685 | 9515 | 13394 | 20570 | 20541 | 17740 | 24944 | 76790 | 47137 | 49208 |
| Class I LINE I | 766115 | 11651 | 17642 | 11762 | 11609 | 21983 | 33791 | 51836 | 51812 | 84502 |
| Class I SINE | 41969 | 9980 | 11414 | 9444 | 10643 | 13526 | 12603 | 27006 | 11310 | 14890 |
| Class II TIR Tc1/Mariner | 890941 | 49626 | 38188 | 41245 | 55210 | 61335 | 93028 | 107075 | 104687 | 130420 |
| Class II MITE | 14346 | 3826 | 4452 | 4661 | 5026 | 7772 | 11072 | 8859 | 7327 | 3919 |
| Unknown | 68696 | 2698 | 3200 | 1645 | 3322 | 8819 | 5056 | 2942 | 1208 | 809 |
| **TE content (% genome)** | **11,15** | **1,56** | **1,00** | **1,18** | **1,49** | **1,60** | **2,38** | **2,00** | **1,47** | **1,64** |

**Figure S2a: Distribution of TE categories of class I and class II main orders and super families in *M. oryzae* 7015**

|  | 70-15 | FR13 | GY11 | PH14 | TH12 | TH16 | US71 | BR32 | CD156 | BR29 |
| --- | --- | --- | --- | --- | --- | --- | --- | --- | --- | --- |
| TE content (% genome) | 11,15 | 1,56 | 1,00 | 1,18 | 1,49 | 1,60 | 2,38 | 2,00 | 1,47 | 1,64 |

**Figure S2b: Distribution of *M. oryzae* 70-15 TE families according to TE space in the 9 *Magnaporthe* isolates**

Thirty seven TE reference families were clusterized into 28 groups. The percentage of TE genome coverage (according to total of TE space) of each group is compared to that of *M. oryzae* 70-15. Values are z-scores of TE content percentage. Data are standardized per group and centered on the reference *M. oryzae* 70-15. Dark orange corresponds to TE over represented with respect to *M. oryzae* 70-15. Dark blue corresponds to TE under represented with respect to *M. oryzae* 70.15.
