# Supplementary material for: Deciphering Genome Content and Evolutionary Relationships of Isolates from the Fungus Magnaporthe oryzae Attacking Different Host Plants
Source: Genome Biol Evol. 2015 Oct 9;7(10):2896–912. doi: 10.1093/gbe/evv187 (PMC4684704; doi:10.1093/gbe/evv187)
Supplement: Supplementary Data [file supp_evv187_Chiapello-SupS6.docx]

**Supplementary S6**. Diversity evaluation and recombination detection in *Magnaporthe oryzae*.

Diversity evaluation and recombination detection in *Magnaporthe oryzae*. We analyzed diversity and recombination in coding regions of *Magnaporthe oryzae* orthologs and in coding region and genomic alignments of the 6 *Magnaporthe oryzae* isolates of the rice lineage. The table includes: the total number of sites (total #sites in millions of positions) and the total number of segments (#segments) in the analyzed alignments, the observed diversity (in %, measured by the Pi index), the mean number of Informative Sites (mean #IS) per alignment and the number of alignments exhibiting significant p-values according to the PHI test (Pairwise Homaplasy Index).
